# Genetic Determinants of Metabolism and Benign Prostate Enlargement: Associations with Prostate Volume

**DOI:** 10.1371/journal.pone.0132028

**Published:** 2015-07-09

**Authors:** Ayush Giri, Todd L. Edwards, Saundra S. Motley, Susan H. Byerly, Jay H. Fowke

**Affiliations:** 1 Institute for Medicine and Public Health, Vanderbilt Epidemiology Center, Vanderbilt University Medical Center, Nashville, TN, United States of America; 2 Vanderbilt Genetics Institute, Vanderbilt University, Nashville, TN, United States of America; 3 Division of Epidemiology, Department of Medicine, Vanderbilt University Medical Center, Nashville, TN, United States of America; 4 Department of Surgical Urology, Vanderbilt University Medical Center, Nashville, TN, United States of America; Innsbruck Medical University, AUSTRIA

## Abstract

Prostate enlargement leading to clinical benign prostatic hyperplasia (BPH) is associated with metabolic dysregulation and obesity. The genetic basis of this association is unclear. Our objective was to evaluate whether single nucleotide polymorphisms (SNPs) previously associated with metabolic disorders are also associated with prostate volume (PV). Participants included 876 men referred for prostate biopsy and found to be prostate cancer free. PV was measured by transrectal ultrasound. Samples were genotyped using the Illumina Cardio-MetaboChip platform. Multivariable adjusted linear regression models were used to evaluate SNPs (additive coding) in relation to natural-log transformed (log) PV. We compared SNP-PV results from biopsy-negative men to 442 men with low-grade prostate cancer with similar levels of obesity and PV. Beta-coefficients from the discovery and replication samples were then aggregated with fixed effects inverse variance weighted meta-analysis. SNP rs11736129 (near the pseudo-gene *LOC100131429*) was significantly associated with log-PV (beta: 0.16, p-value 1.16x10^-8^) after adjusting for multiple testing. Other noteworthy SNPs that were nominally associated (p-value < 1x10^-4^) with log-PV included rs9583484 (intronic SNP in *COL4A2*), rs10146527 (intronic SNP in *NRXN3*), rs9909466 (SNP near *RPL32P31*), and rs2241606 (synonymous SNP in *SLC12A7*). We found several SNPs in metabolic loci associated with PV. Further studies are needed to confirm our results and elucidate the mechanism between these genetic loci, PV, and clinical BPH.

## Introduction

A highly prevalent condition in aging men, benign prostatic hyperplasia (BPH) is the non-malignant proliferation of the epithelial and stromal cells in the prostate gland [1]. It is often diagnosed in the presence of enlarged prostate and bladder outlet obstruction leading to lower urinary tract symptoms (LUTS)[1]. In the year 2000, there were approximately 4.5 million physician visits with a primary diagnosis of BPH, and approximately 1.3 billion dollars were spent in healthcare costs relating to BPH care [2]. Given the aging population in the US and the high prevalence of BPH, costs of treatment are likely to remain high.

The etiology of BPH is not fully understood. In the last decade, interest has increased for metabolic syndrome and markers of metabolism as potential risk factors for BPH and LUTS [3–7]. Several studies have found a positive association between measures of obesity (body mass index, waist to hip ratio, waist circumference) and a number of outcomes related to BPH including increased prostate volume (PV), LUTS, and BPH treatment [8–14]. A recent secondary analysis of the Reduction by DUtasteride of prostate Cancer Events (REDUCE) trial showed that not only was obesity positively associated with prostate volume, but that obesity also attenuated the effects of dutasteride to reduce PV [15]. Factors associated with increased risk for cardiovascular disease, such as elevated fasting blood glucose levels, insulin levels, and diabetes [4,5,13,14,16–18], dysregulated lipids [16,18,19], and inflammation [20–24] have been associated with increased risk for BPH and/or LUTS. Similarly, meta-analyses have reported that moderate regular alcohol consumption [25] and physical exercise [26] are inversely associated with BPH and risk for cardiovascular disease.

While genetic susceptibility to metabolic dysregulation may contribute to BPH progression, few studies have evaluated genetic susceptibility to BPH. A study among twins found high concordance rates for benign prostate disease, suggesting a hereditary influence [27]. Most studies examining genetic markers for association with BPH have evaluated a few candidate genes involved in androgen activity, including androgen receptors and steroid alpha-reductase type II (SRD5A2). However, results from these studies are not conclusive [28–31], and, much like prostate cancer (PC), it is unclear that genetic variability in androgen activity contributes to PV or BPH progression. In contrast, there do appear to be shared associations between BPH and PC susceptibility, as a recent study evaluating 14 SNPs previously associated with PC found SNPs near *IRX4*, *ITGA6*, and *RFX6* genes were also associated with increased BPH risk or increased BPH aggressiveness [32]. The exact mechanisms of how these genes may promote PC or BPH are not clear. To date, there has been no systematic evaluation of the genome in relation to BPH.

We used the Illumina Cardio-MetaboChip to evaluate associations between PV and over 100,000 genetic polymorphisms in genes or genomic regions previously associated with anthropomorphic and metabolic traits [33]. Considering the consistent evidence for an association between metabolic dysregulation and BPH, but the limited number of studies investigating genetic risk factors for BPH, our study provides the most thorough evaluation to date of the hypothesis that BPH and metabolism share common genetic determinants.

## Methods

Participants for this study were participants of the Nashville Men’s Health Study (NMHS), a multi-center, rapid-recruitment protocol targeting men seeking a diagnostic prostate biopsy. Briefly, among men referred for prostate biopsy to VUMC and to the Tennessee Valley Veterans Administration in Nashville between January 1, 2006 and March 30, 2011, men who were ≥40 years of age, had English proficiency sufficient for informed consent, no prior diagnosis of PC, no prior prostate surgery, and reported no current use of androgen supplementation were considered eligible for participation. Institutional review boards at Vanderbilt University Medical Center (VUMC) and at the Tennessee Valley Veterans Administration (Nashville, TN) approved all study aspects including and not limited to recruitment, data collection protocols and written informed consent procedures. All study participants provided written informed consent. Recruitment procedures were conducted prior to the prostate biopsy and transrectal ultrasound (TRUS) procedures to prevent selection bias during data collection associated with knowledge of outcome.

Trained research staff collected information on demographic characteristics and lifestyle factors, and measured anthropometric traits including height, weight, hip and waist circumference, which were used to calculate body mass index (BMI) and waist-to-hip ratio (WHR). Blood samples were genotyped with the Illumina Cardio-MetaboChip [33], which is a genotyping array of 217,695 SNPs within 257 genomic regions implicated in 23 metabolic traits including obesity, fasting glucose, blood lipids and several cardiovascular traits. Information on PC diagnoses, tumor aggressiveness—as reported by total Gleason score, and prostate volume (ml)—as determined by TRUS, were abstracted from medical charts. A single pathologist then reviewed over 90% of the biopsies. Men with a negative biopsy and without indication of PC, pathology suspicious of PC, or high-grade prostatic intraepithelial neoplasia were considered without PC at that time and eligible for our analysis. Patients with positive result from prostate biopsy and confirmed as having non-metastatic PC (PC as primary cancer diagnosis) at recruitment were considered cases. PC cases with a total Gleason score of 7 or more were defined as high-grade PC cases.

A total of 969 eligible men free of PC with DNA availability were randomly selected for genotyping and served as our primary research group to examine the relationship between SNPs and prostate volume. We also wanted to investigate the consistency of any signals in a separate group of 520 men with low-grade PC. These men were recruited from the same recruitment protocol as the biopsy-negative men, with identical data and bio-specimen protocols. The rationale for the comparison is that low-grade and localized PC lesions are common, and with the exception of the detection of a localized tumor, men without PC at biopsy and men with low-grade PC are similar with regard to prostate size, prostate-specific antigen (PSA) levels, BMI, and the prevalence of CVD and other obesity-related comorbidities. We intentionally did not include high-grade PC cases from the validation sample because genetic factors leading to advanced PC are likely to be distinct from those involved in prostate enlargement, and men with high-grade PC had significantly smaller prostate volume and higher PSA levels than men with either low-grade PC or men without PC at biopsy. In other words, elevated PSA levels in men diagnosed with high-grade PC are likely a consequence of the PC, whereas elevated PSA levels among men with low-grade PC are likely, or in large part, a consequence of increasing PV, similar to men with elevated PSA and without PC [34,35].

### Data quality control

Nine hundred and forty one men without PC and 459 low-grade PC cases were successfully genotyped. Post genotype-calling quality control (QC) procedures were conducted using the software package PLINK [36]. Individuals with sample call rates <95% (36 PC-free men, 9 PC cases) were first excluded. In the remaining samples, SNPs were excluded if genotyping efficiency was less than 98% (15,716 SNPs). Identity-by-descent analysis of pruned independent SNPs in linkage equilibrium was conducted to estimate cryptic relatedness between samples. We excluded 15 samples (10 PC-free men and 5 PC cases) as they were either monozygotic twins or inadvertent duplicate samples. Identity-by-descent analysis further revealed 10 sample pairs with detectable cryptic relatedness at the first cousin level or higher. We removed 10 samples (9 PC-free men and 1 PC case) due to cryptic relatedness. We additionally removed SNPs that deviated from Hardy-Weinberg equilibrium at the p-value threshold < 1x10^-6^ (4,712 SNPs). We further excluded 38 individuals (30 PC-free men and 2 PC cases) as they did not have data available for prostate volume. Our final analysis consisted of a discovery sample of 876 men without PC at biopsy and a validation sample of 442 men with low-grade PC, for whom data were also available for both prostate volume and genotype information. We limited our investigation to SNPs that had minor allele frequencies of 5% or more, which left us with a total of 100,559 SNPs available for primary data analysis. We used principal component analysis with EIGENSOFT to evaluate the presence of population substructure in our study sample. We derived 10 principal components after LD-pruning our dataset to include 39,980 independent markers with minor allele frequency ≥0.05. A plot representing the first two principal components for our samples along with reference populations from the International HapMap Project has been published previously [37].

### Statistical analysis

We transformed the continuous PV (ml) variable using the natural-log function to approximate the normal distribution. We compared the characteristics of men without PC and men with low-grade PC with the student’s t-test with unequal variances for comparison of group means, Mann-Whitney rank sum test for the comparison of group medians, and Pearson’s chi-squared test for categorical variables. To ensure that the associations between major risk factors for PV in our study sample are comparable to that of other studies that have evaluated PV as a dependent variable, and also to ensure that the associations are similar among men without PC and men with low-grade PC, we used linear regression to evaluate the relationship between important covariates and log-transformed PV.

In primary analyses we used linear regression in PLINK to evaluate the relationship between SNPs (assuming an additive SNP coding model) and natural-log transformed PV in two models 1) a minimally adjusted model including age and 10 principal components; 2) a fully adjusted model including age, height, BMI and 10 principal components in men without PC and men with low-grade PC, separately. Results from both models were similar; we present results from fully adjusted models. We first analyzed men without PC and men with low-grade PC separately, and then to summarize results from these two groups we performed an inverse variance weighted fixed effect meta-analysis of the beta-coefficients from our models considering men without PC and men with low-grade PC. We present beta-coefficients and standard errors as change of log-PV per minor allele. To account for multiple-testing we chose to estimate the family-wise error rate (FWER) in our study. We first used simpleM to estimate the effective number of independent tests [38]. Briefly, simpleM calculates the effective number of tests by accounting for correlations between the SNPs tested. From the 100,538 SNPs we tested, we estimated the effective number of tests to be 64,858. We then divided 0.05 by this number to establish a p-value threshold (7.71x10^-7^) that considers the multiple tests in our study. We evaluated genomic inflation with a quantile-quantile plot (S1 Fig).

To separate the genetic vs. non-genetic contributions of height, BMI and WHR on prostate volume, we created genetic risk scores (GRS) for height, BMI, and WHR from SNPs previously identified by GIANT and CHARGE consortium analyses to be associated with BMI [39], WHR [40], and height [41]. Of the 32 reported SNPs for BMI, 14 reported SNPs for WHR and 180 SNPs reported for height, our post QC dataset had 24 SNPs, 14 SNPs and 151 SNPs available for the creation of BMI, WHR and height GRSs, respectively. We applied two weighting schemes assuming an additive model, to create two different GRSs for each trait in consideration: 1) equal weighting for SNPs, where the score per individual is simply a count of the number of risk-increasing alleles for the given individual; 2) beta-coefficient weighting for SNPs, where the score per individual is a weighted sum of the number of risk-increasing alleles for the given individual, and the weight for each SNP is the effect estimate associated with the given anthropomorphic trait involved (BMI, WHR, or height) as previously reported in the literature [39–41]. The average genotyping rate for the SNPs included in the analysis was >99%. Individuals for whom information on a given SNP was missing, GRSs were prorated by dividing by the number of contributing alleles by the number of non-missing SNP genotypes per individual.

We then used STATA to regress natural-log transformed values of prostate volume against un-weighted and weighted genetic risk scores for height, BMI and WHR in linear regression models that adjusted for age and 10 principal components of ancestry, and with or without traits of interest (height, BMI, WHR, as applicable).

## Results

Distribution of characteristics in men without PC and men with low-grade PC are presented in Table 1. Age, BMI, diabetes status, and use of statins or NSAIDs were similar between biopsy-negative men and men with low-grade PC. PV ranged from 2.00 ml to 319 ml in men without PC, and 5.46 ml to 167 ml among men with low-grade PC. Although median prostate volume was approximately 8 ml lower in men with low-grade PC compared with men without PC (p-value <0.001), a substantial number of men in both groups as a PV > 40 ml (44.3%), and PSA levels were not significantly different across groups.

**Table 1:** Study Characteristics of Men without Prostate Cancer (PC) and with Low-grade PC: the Nashville Men’s Health Study.

Variable	No PC (n = 876)	Low-Grade PC (n = 442)	
	Mean (SD)/Median (IQR)	Mean (SD)/Median (IQR)	p-value*
**Age, y**	64.3 (8.6)	64.8 (8.3)	0.394
**Height, cm**	175.2 (6.9)	175.3 (6.9)	0.839
**BMI, kg/m^2^**	29.0 (4.6)	28.6 (4.4)	0.120
**WHR**	1.0 (0.1)	1.0 (0.1)	0.396
**Prostate volume, ml**	45.9 (33.0–64.3)	37.7 (28.5–52.5)	<0.001
**PSA, ng/ml**	5.1 (4.0–6.9)	5.1 (4.2–7.1)	0.100
	N (%)	N (%)	
**Diabetes status**			0.576
No	759 (86.6)	378 (85.5)	
Yes	117 (13.4)	64 (14.5)	
**Statin use**			0.192
No	540 (61.6)	256 (57.9)	
Yes	336 (38.4)	186 (42.1)	
**NSAID use**			0.918
No	399 (45.6)	200 (45.3)	
Yes	477 (54.4)	242 (54.7)	

*p-values from student’s t-test with unequal variances (for comparing group means), or from Mann-Whitney test (for comparing group medians), or Pearson’s chi-squared test (for categorical variables). SD = Standard deviation; IQR = Inter quartile range

Non-genetic predictors of PV were similar across diagnostic groups. Age, height, BMI, and statin use were all independently and positively associated with increased prostate volume in participants without a PC diagnosis and participants with low-grade PC (Table 2). WHR and diabetes were also associated with PV, but these associations were lost after controlling for age, BMI and other listed covariates. Furthermore, NSAID use was not associated with PV.

**Table 2:** Determinants of Prostate Volume (Natural Log-transformed) in Men without Prostate Cancer (PC) and with Low-grade PC: the Nashville Men’s Health Study.

Variable	No PC (n = 876)			Low-Grade PC (n = 442)		
	Beta	SE	p-value	Beta	SE	p-value
**Age, y**						
Unadjusted	0.017	0.002	<0.001	0.015	0.003	<0.001
Adjusted^a^	0.020	0.002	<0.001	0.019	0.002	<0.001
**Height, cm**						
Unadjusted	0.002	0.002	0.327	0.008	0.003	0.012
Adjusted^a^	0.007	0.002	0.002	0.011	0.003	<0.001
**BMI, kg/m^2^**						
Unadjusted	0.019	0.004	<0.001	0.0217	0.005	<0.001
Adjusted^a^	0.023	0.004	<0.001	0.027	0.005	<0.001
Adjusted^c^	0.024	0.004	<0.001	0.025	0.006	<0.001
**WHR** *						
Unadjusted	0.094	0.023	<0.001	0.125	0.031	<0.001
Adjusted^b^	0.066	0.023	0.004	0.108	0.03	<0.001
Adjusted^c^	-0.012	0.026	0.662	0.019	0.035	0.584
**Diabetes status**						
Unadjusted	0.100	0.051	0.050	0.108	0.062	0.083
Adjusted^a^	-0.013	0.049	0.793	-0.034	0.062	0.586
**Statin use**						
Unadjusted	-0.004	0.036	0.911	-0.027	0.045	0.543
Adjusted^a^	-0.066	0.033	0.050	-0.114	0.042	0.007
**NSAID use**						
Unadjusted	0.008	0.035	0.818	0.062	0.044	0.160
Adjusted^a^	-0.042	0.033	0.208	0.016	0.042	0.689

Unadjusted models: natural log-transformed PV regressed against the variable of interest only

^a^Independent variables in the model: Age, Height, BMI and 10 genetic principal components

^b^Independent variables in the model: Age, Height, WHR and 10 genetic principal components

^c^Independent variables in the model Age, Height, BMI, WHR and 10 genetic principal components

*Betas represent per 0.1 unit increase in WHR

A total of 100,538 SNPs with minor allele frequencies of 5% or higher were available for our single variant analyses. We estimated the association between each SNP and PV in multivariable linear regression models adjusting for age, height, BMI, and 10 principal components summarizing ancestry. Analyses were conducted within each group, and then inverse variance weighted fixed effect meta-analysis was used to combine beta coefficients from the two clinical groups. There were 5 independent loci associated with PV from the meta-analysis at a p-value threshold of less than 1x10^-4^ (Table 3). SNP rs11736129, near the *LOC100131429* gene, was the only statistically significant SNP after accounting for multiple testing (Beta 0.16, p-value 1.16x10^-8^), and heterogeneity in the rs11736129 and PV association was low between the two clinical groups. (I^2^ = 11%), indicating a similar association within each group. SNP rs9583484, located in the intronic region of the *COL4A2* gene was nominally associated with PV (Beta -0.11, p-value 1.01x10^-05^), with a stronger association among biopsy-negative men (I^2^ = 51%). Additionally, two SNPs near *NRXN3* (rs10146527, Beta = -0.08, p-value = 3.49x10^-05^; rs2202167, Beta = 0.08, p-value = 3.49x10^-05^), a SNP near *RPL32P31* (rs9909466, Beta = 0.09, p-value = 5.88x10^-05^) and a synonymous polymorphism in the *SLC12A7* exon (rs2241606, Beta = 0.07, p-value = 8.51x10^-05^) were nominally associated with log-PV at the p-value threshold of < 1x10^-04^.

**Table 3:** Genetic Determinants of Prostate Volume at p<1x10^-4^ from Meta-analysis Combining Results across Diagnostic Groups: the Nashville Men’s Health Study.

					No PC				Low-Grade PC				Fixed effect meta-analysis		
SNP	CHR	BP	On/Nearby Genes	MA/RA	MAF	Beta	SE	p-value	MAF	Beta	SE	p-value	Beta	p-value	I^2^
rs11736129	4	139605076	LOC100131429	G/C	0.12	0.14	0.04	1.14x10^-04^	0.10	0.20	0.05	1.97x10^-05^	0.16	1.16x10^-08^	11%
rs9583484	13	109764350	COL4A2*/COL4A1	T/G	0.17	-0.13	0.03	1.69x10^-05^	0.17	-0.06	0.04	9.60x10^-02^	-0.11	1.01x10^-05^	51%
rs10146527	14	78569603	NRXN3*	C/T	0.38	-0.08	0.02	7.35x10^-04^	0.39	-0.07	0.03	1.70x10^-02^	-0.08	3.49x10^-05^	0%
rs2202167	14	78569378	NRXN3*	A/C	0.38	-0.08	0.02	8.39x10^-04^	0.39	-0.07	0.03	1.64x10^-02^	-0.08	3.80x10^-05^	0%
rs9909466	17	76113835	RPL32P31	C/G	0.19	0.08	0.03	4.43x10^-03^	0.21	0.10	0.04	4.63x10^-03^	0.09	5.88x10-^05^	0%
rs2241606	5	1110615	SLC12A7**	A/G	0.39	0.07	0.03	2.07x10^-03^	0.39	0.07	0.03	1.55x10^-02^	0.07	8.51x10^-05^	0%

CHR = Chromosome; SNP = Single Nucleotide Polymorphism MA = Minor Allele; RA = Referent Allele; MAF = Minor Allele Frequency; I^2^ = amount of heterogeneity between groups not explained due to chance; Table sorted by Fixed effects P value;

*SNP is on the intron region of the gene

**SNP is on the exon region of the gene

We next identified loci associated with log-PV within each clinical group at a p-value threshold of <1x10^-4^ (Table 4). Among men with a negative prostate biopsy, SNPs meeting the nominal significance level included rs10400014 (Beta = 0.12, p-value = 2.05x10^-5^) near *ZEB1 and ARHGAP12*, and rs12662869 (Beta = -0.10, p-value = 8.64x10^-5^) in and intron of *SLC17A1/A4*. In men with low-grade PC, PV was associated with SNPs in the intronic regions of *PKP2*, *AKAP13*, and SNPs near *LOC100131429*, *AGTR1*, and *ANAPC1*.

**Table 4:** MetaboChip SNPs Nominally Associated with Natural-log Transformed Prostate Volume at p <1x10^-4^ in Men without Prostate Cancer (PC) and Men with Low-grade PC: the Nashville Men’s Health Study.

SNP	CHR	BP	On/Nearby Genes	MA/RA	MAF	Beta	95% CI	p-value	n
**No PC**									
rs10400014	10	32015299	ZEB1, ARHGAP12	C/A	0.24	0.12	(0.06, 0.17)	2.05x10^-05^	875
rs9416979	10	32014833	ZEB1, ARHGAP12	G/A	0.47	0.10	(0.05, 0.14)	3.01x10^-05^	876
rs11593009	10	32014952	ZEB1, ARHGAP12	T/A	0.20	0.12	(0.06, 0.17)	6.37x10^-05^	876
rs1418363	10	32015069	ZEB1, ARHGAP12	A/G	0.44	0.09	(0.05, 0.14)	8.38x10^-05^	876
rs12662869	6	25892460	SLC17A1*, SLC17A4	A/C	0.31	-0.10	(-0.15, -0.05)	8.64x10^-05^	876
rs3034359	6	25900692	SLC17A1*, SLC17A4	T/G	0.30	-0.10	(-0.15, -0.05)	9.36x10^-05^	871
**Low-grade**									
rs11615932	12	32844340	PKP2*	A/G	0.06	-0.28	(-0.40, -0.15)	1.92x10^-05^	442
rs1483581	15	83865913	AKAP13*/XR_243224.1*	G/A	0.24	-0.14	(-0.21, -0.08)	2.10x10^-05^	440
rs7640795	3	149785644	AGTR1	C/T	0.26	-0.14	(-0.21, -0.08)	2.53x10^-05^	442
rs16940997	15	83873130	AKAP13*	C/A	0.24	-0.14	(-0.20, -0.07)	4.98x10^-05^	442
rs12373588	2	112182736	LOC100133059/ANAPC1	G/T	0.43	0.11	(0.06, 0.17)	8.65x10^-05^	442

CHR = Chromosome; SNP = Single Nucleotide Polymorphism MA = Minor Allele; RA = Referent Allele; MAF = Minor Allele Frequency; Beta Coefficient from linear regression model evaluating natural log transformed prostate volume as a continuous dependent variable, while adjusting for age (continuous), body mass index (continuous), height (continuous), and 10 genetic ancestry principal components.

*SNP is on the gene; but is on the intron region

As anthropomorphic traits such as height and measures of obesity are consistently associated with prostate enlargement, we additionally investigated the associations between GRSs of height, WHR and BMI in relation to log-PV in each group (Table 5). Both un-weighted and weighted GRSs for height, BMI and WHR were not associated with log-prostate volume, with or without adjustment for its corresponding anthropometric trait.

**Table 5:** Natural Log-transformed Prostate Volumes Regressed against Un-weighted and Weighted Genetic Risk Scores (GRS) for Height, WHR and BMI with and without Adjustment for Height, WHR and BMI, Respectively, Among Men without Prostate Cancer (PC) and Men with Low-grade PC: the Nashville Men’s Health Study.

Prostate Status	Un-weighted GRS	Beta	95% CI	p-value	Weighted GRS	Beta	95% CI	p-value
**No PC** (N = 876)	**Height-GRS**				**Height-GRS**			
	^a^Model 1	-0.001	(-0.004, 0.004)	0.793	^a^Model 1	0.024	(-0.091, 0.140)	0.68
	^b^Model 2	-0.002	(-0.006, 0.002)	0.405	^b^Model 2	-0.013	(-0.130, 0.105)	0.883
	**WHR-GRS**				**WHR-GRS**			
	^c^Model 3	-0.002	(-0.015, 0.012)	0.843	^c^Model 3	-0.025	(-0.227, 0.176)	0.805
	^d^Model 4	-0.001	(-0.014, 0.013)	0.902	^d^Model 4	-0.021	(-0.221, 0.180)	0.841
	**BMI-GRS**				**BMI-GRS**			
	^e^Model 5	-0.006	(-0.017, 0.005)	0.267	^e^Model 5	-0.016	(-0.068, 0.035)	0.534
	^f^Model 6	-0.006	(-0.017, 0.005)	0.311	^f^Model 6	-0.009	(-0.059, 0.042)	0.728
**Low-Grade PC** (N = 442)	**Height-GRS**				**Height-GRS**			
	^a^Model 1	-0.004	(-0.015, 0.006)	0.384	^a^Model 1	0.048	(-0.101, 0.197)	0.528
	^b^Model 2	-0.001	(-0.011, 0.010)	0.953	^b^Model 2	-0.008	(-0.158, 0.142)	0.919
	**WHR-GRS**				**WHR-GRS**			
	^c^Model 3	0.001	(-0.015, 0.017)	0.89	^c^Model 3	0.004	(-0.246, 0.255)	0.973
	^d^Model 4	0.001	(-0.015, 0.017)	0.873	^d^Model 4	0.032	(-0.215, 0.279)	0.8
	**BMI-GRS**				**BMI-GRS**			
	^e^Model 5	-0.001	(-0.015, 0.012)	0.87	^e^Model 5	-0.001	(-0.064, 0.061)	0.967
	^f^Model 6	-0.001	(-0.012, 0.014)	0.878	^f^Model 6	0.016	(-0.044, 0.077)	0.598

Un-weighted genetic risk scores and weighted genetic risk scores for height, waist to hip ratio and body mass index were created as described in the methods section. These risk scores were then regressed against natural log-transformed prostate volume while adjusting for a number of covariates, including and excluding the anthropometric trait for which the genetic risk score was created.

^a^Model 1: Adjusted for Age, BMI, and 10 genetic ancestry principal components

^b^Model 2: Adjusted for Age, BMI, Height and 10 genetic ancestry principal components

^c^Model 3: Adjusted for Age, Height and 10 genetic ancestry principal components

^d^Model 4: Adjusted for Age, Height, WHR and 10 genetic ancestry principal components

^e^Model 5: Adjusted for Age, Height and 10 genetic ancestry principal components

^f^Model 6: Adjusted for Age, Height, WHR and 10 genetic ancestry principal components

## Discussion

Metabolic syndrome and its components have been hypothesized to increase risk for BPH and LUTS, however it is not clear if these associations are at least in part mediated by genetic susceptibility to the metabolic syndrome, and if so, by which specific variants. Hypothesizing that genetic variants related to the metabolic syndrome may also be positively associated with BPH, we evaluated SNPs throughout the genome that have been implicated with several metabolic disorders in relation to prostate volume in men without PC and men with low-grade PC. As the first study to systematically assess this hypothesis, we report a genome-wide significant association and also provide preliminary evidence for several additional loci putatively associated with prostate volume.

rs11736129, the most statistically significant result in our analysis, lies approximately 13 kilo-bases downstream of the pseudo-gene *LOC100131429*, which bears sequence similarity to the armadillo repeat containing 1 gene (*ARMC1*) which encodes a metal ion binding protein. While pseudo-genes lack coding potential due to the presence of various mutations such as premature stop codons and frame shifts, unprocessed pseudo-genes like the *LOC100131429* may be transcribed. The SNP rs11736129 is annotated by ENCODE to lie within an enhancer histone mark, and thus may be an expression quantitative trait locus [42]. As has been demonstrated for the *MYLKP1* pseudo-gene and its functional counterpart, the expression of the non-coding RNA of a pseudo-gene may inhibit the expression of the functional gene by decreasing its mRNA stability [43]. RNA expression analysis reports from Genecards show that the *LOC100121429* pseudo-gene and the *ARMC1* gene are expressed in normal human prostate tissues [44]. The *ARMC1* gene was associated with childhood obesity in a Hispanic population [45], however the biological mechanisms relating *ARMC1* pseudo-gene and BPH are speculative and require confirmation at this point. SLC7A11 is the closest protein-coding gene near rs11736129, which is approximately 250KB from the SNP. The gene product of SLC7A11 is a component of an anionic antiporter transport system which regulates cysteine and glutamine transport. This transport system, also known as the xCT antiporter system, has been proposed as a drug intervention target for cancers such as common triple-negative breast cancer [46], glioma [47] and pancreatic cancer [48].

Among loci for which evidence was suggestive, we found two polymorphisms in the *NRXN3* associated with PV. *NRXN3* gene product plays important role in cell adhesion, is expressed in the prostate tissue [49], and has been shown to be differentially overexpressed in an androgen dependent PC cell line compared with an androgen independent PC cell line [50]. Interestingly, *NRXN3* was significantly associated with waist-circumference in a large analysis by the CHARGE consortium [51], with BMI by the GIANT consortium [39] and clinical measures of overweight and obesity [52]. Similar evidence was observed at the SNP rs9583484 in the intronic region of the Type IV collagen *COL4A2* gene, a gene providing the major structural component of basement membranes. *COL4A2* is expressed in normal prostate tissues, induced by androgens, and the C-terminal portion of the *COL4A2* gene is thought to inhibit angiogenesis [53]. *COL4A2* has been suggested as a biomarker for screening BPH [54]. Pritchard and colleagues suggest that type IV collagen genes, including *COL4A1* and *COL4A2* may be over-expressed, and *COL3A1* and *COL5A2* expression may be repressed by androgen exposure [53], suggesting an alternative pathway by which androgen activity may influence BPH progression. Other loci of interest included a polymorphism in the *RPL32P31* pseudogene and polymorphisms in the solute carrier family of genes (*SLC12A7*, *SLC12A1*) involved in the transport of sodium and other inorganic compounds across the cell membrane and the sodium/potassium channel (*SLC17A1*).

As the first study to systematically evaluate *a priori* determined metabolic genetic variants throughout the genome in relation to PV, our results are preliminary and need to be replicated by other independent studies before drawing any definitive conclusions. Assuming a minor allele frequency of 0.10, a conservative p-value threshold of (5x10^-7^; assuming 100,000 independent tests), and a sample size of 1300 participants (assuming both PC-free men and low-grade PC cases together), we had approximately 83% power to detect a beta-coefficient of 0.15. The effect estimates that we observed for the most part were less than 0.15, and we were likely underpowered to detect these smaller associations. With the exception of SNP at *LOC100131429* that reached genome-wide significance (p-value 1.16x10^-8^), the associations for all of the other SNPs reported here are not statistically significant after adjustment for multiple-comparisons.

We note several limitations of this study. Firstly, our analysis is limited to the evaluation between SNPs and total prostate volume. We did not have information on transitional zonal volume; availability of this information would have provided for a more refined analysis as transitional zone has been shown to be a better predictor of BPH severity than total prostate volume [55]. Understanding that prostate enlargement is one of the many components involved in the diagnosis of BPH, we acknowledge the study’s inability to draw associations between SNPs and BPH/LUTS severity. While we administered a standardized set of questions which assessed the International Prostate Symptom Score (I-PSS), this information was missing on approximately 60% of the participants in this study. Although the I-PSS provides an assessment of the severity of BPH, it has been shown that scores are correlated with not just symptom severity but depends on many factors including patient awareness and socioeconomic status [56–58]. With this in mind, the I-PSS may not be a strong candidate for testing associations with germ line variations in the genome. Instead, we elected to evaluate the relationship between SNPs and prostate volume, which is a reliably quantifiable component of BPH which is less prone to misclassification. Secondly, the pathology reports collected on the prostate tissues were limited to the assessment of prostate cancer rather than inflammation characteristics including infiltration of immune cells in the prostate. Therefore, we are not able to comment on the relationship between SNPs and inflammation in the prostate. Thirdly, our assessment of metabolic characteristics of the patients was limited to anthropometric assessments and diabetes status. A more detailed inventory of metabolic characteristics would have allowed for an analysis that adjusted for these characteristics. These metabolic characteristics would not be confounders in the association between SNPs and prostate volume but would rather most likely be in the causal pathway. With this regard, we were not able to test whether the associations observed are independent of these comorbidities; however this should not invalidate our current findings that SNPS previously associated with a range of metabolic traits also are associated with increasing PV.

Despite these caveats, our study has several strengths. The measurement of PV and accession of covariates for all participants in the study were taken prior to prostate biopsy, therefore reducing the potential for information bias. We used PC-free men as our discovery sample and men with low-grade PC as our validation sample after first demonstrating that, aside from prostate volume, these two groups had similar body size and clinical characteristics. Obesity may have a separate association with advanced PC [59], and we therefore excluded men with high-grade PC hypothesizing that any association between genetic variability in men with high-grade PC would likely be a consequence of effects on PC rather than prostate size. In contrast, men with low-grade PC are more like men without PC, with many diagnosed with incidental PC as a consequence of PSA testing. These low-grade PC patients have an excellent prognosis, with 5-year survivorship estimated at 100%. Consistent with our hypothesis, we showed that these two groups shared associations between risk factors for prostate enlargement such as age, height, BMI, WHR and statin use. Although the low-grade PC group had a significant 6–8 ml smaller average prostate volume those men without PC, this difference is clinically marginal toward BPH progression, and PSA levels were similar between groups. It is, however, likely that some portion an elevated PSA levels in this low-grade PC was a consequence of concurrent PC as well as prostate enlargement. To address the potential heterogeneity between these groups, we conducted analyses separately on these two groups and then combined the results in a meta-analysis of the beta-coefficients. Results were generally consistent across groups, with low to moderate indication of heterogeneity.

Finally, we evaluated the compound genetic components of metabolism-related traits such as height, BMI and WHR in relation to prostate volume. Height, BMI, and WHR were positively associated with prostate volume. However, our investigation of genetic risk scores for these anthropomorphic traits did not identify a shared genetic component between body size measures and prostate enlargement, suggesting that the association between obesity or height with prostate size is a consequence of endocrine factors or other non-genetic factors.

In conclusion, we identified several genetic loci related to metabolic disorders that were also associated with prostate enlargement. Replication of these loci in additional populations and subsequent functional and mechanistic studies will be needed to further understand the genetic epidemiology of BPH.

## Supporting Information

S1 Fig QQ-PLOT: Meta-analysis for natural log transformed prostate volume beta-estimates among individuals with no prostate cancer and low-grade prostate cancer. Quantile-quantile plot for the meta-analysis p-values from fully adjusted model: adjusted for age, height, BMI and 10 principal components; BMI = body mass index (kg/m^2^). Plot shows the expected p-values under the uniform distribution versus the observed p-values from study. PC = Prostate Cancer.
(TIFF)
