# Supplementary figures and images for: Genetic Determinants of Metabolism and Benign Prostate Enlargement: Associations with Prostate Volume
Source: PLoS One. 2015 Jul 9;10(7):e0132028. doi: 10.1371/journal.pone.0132028 (PMC4497718; doi:10.1371/journal.pone.0132028)

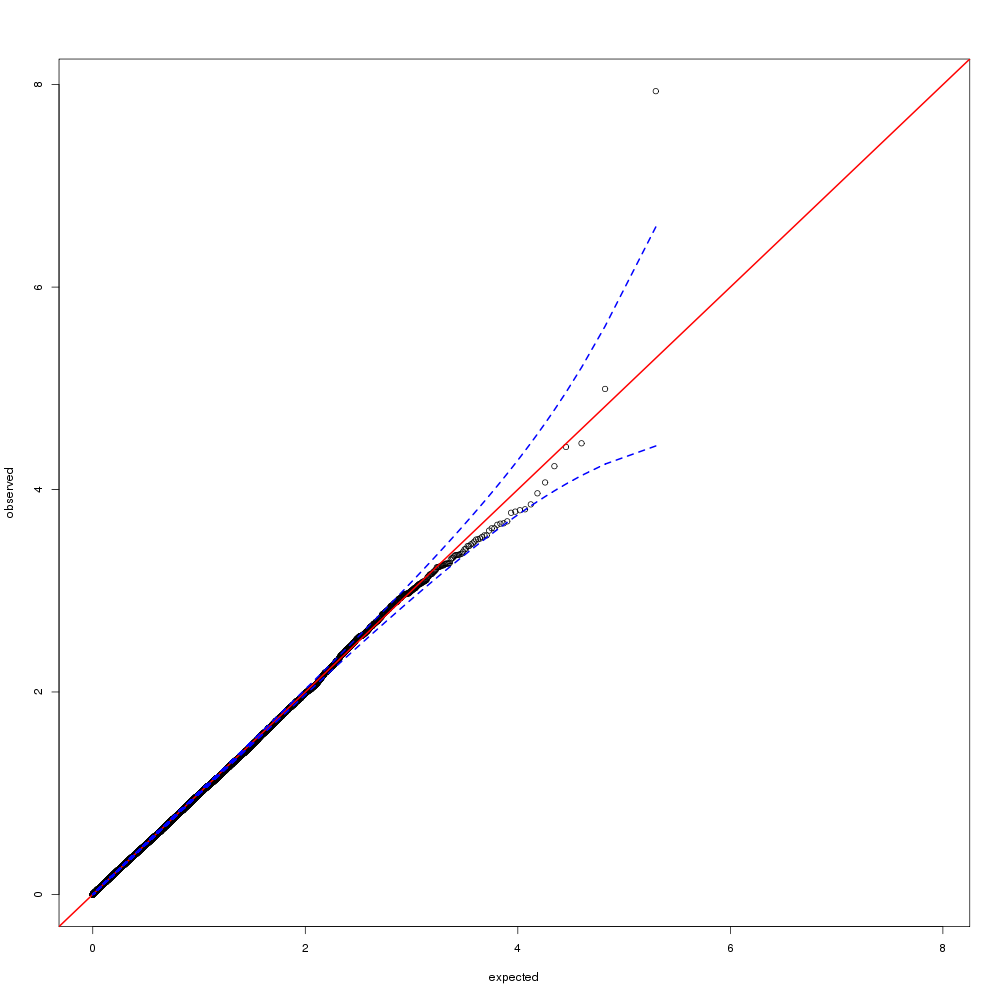

Supplement: S1 Fig — Quantile-quantile plot for the meta-analysis p-values from fully adjusted model: adjusted for age, height, BMI and 10 principal components; BMI = body mass index (kg/m2). Plot shows the expected p-values under the uniform distribution versus the observed p-values from study. PC = Prostate Cancer. (TIFF) [file pone.0132028.s001.tiff]
